# Determinants of Carbapenem-Resistant *Klebsiella pneumoniae*: Clinical Outcomes and Epidemiological Risk Factors in a Single-Center Cohort Dataset †

**DOI:** 10.3390/antibiotics15060621

**Published:** 2026-06-18

**Authors:** Cristiana Ana-Maria Olguța Penea, Violeta Melinte, Claudia Simona Cambrea, Tiberiu Holban, Adelina Maria Radu, Cristina Maria Vacaroiu, Valeriu Gheorghiță

**Affiliations:** 1 ”Agrippa Ionescu” Clinical Emergency Hospital, 011356 Bucharest, Romania; 2 Department of Infectious Diseases, “Carol Davila” University of Medicine and Pharmacy, 050474 Bucharest, Romania; 3 Faculty of Medicine, “Ovidius” University, 900470 Constanta, Romania

**Keywords:** *K. pneumoniae*, carbapenem resistance, CRE colonization, clinical adjudication, Sepsis-3, ICU, antimicrobial stewardship, delayed active therapy, carbapenemase-producing Enterobacterales

## Abstract

**Background:** Carbapenem-resistant *K. pneumoniae* (CRKP) represents a major challenge in hospitalized patients because of its association with healthcare exposure, restricted antimicrobial options, and adverse clinical outcomes. Microbiological isolation alone does not define invasive disease; therefore, clinical interpretation requires separation of colonization, localized infection, invasive infection, and carbapenem-resistant Enterobacterales (CRE)-associated sepsis. This study evaluated epidemiological features, resistance phenotypes, treatment adequacy, and clinical outcomes among hospitalized adults with *K. pneumoniae* isolates, using a clinical framework that distinguishes colonization from active infection and invasive disease. **Methods:** This single-center retrospective observational cohort study included 157 consecutive adults admitted between January and July 2025 to a tertiary-care hospital with at least one microbiologically confirmed *K. pneumoniae* isolate recovered from clinical specimens and/or CRE surveillance rectal swabs. Isolates were assigned hierarchically to four mutually exclusive phenotypic groups: carbapenem-susceptible *K. pneumoniae* (CSKP), extended-spectrum beta-lactamase (ESBL)-producing carbapenem-susceptible *K. pneumoniae* (ESBL), carbapenem-resistant non-carbapenemase-producing *K. pneumoniae* (CRKP), and carbapenemase-producing *K. pneumoniae* (CP-KP). A prespecified secondary analysis compared carbapenem-resistant isolates (CRKP + CP-KP) with non-carbapenem-resistant isolates (CSKP + ESBL). Clinical adjudication distinguished colonization-only cases, non-invasive infection, bloodstream infection, device-associated infection, and CRE-associated sepsis; ventilator-associated pneumonia (VAP) was considered when source data allowed reliable attribution. Sepsis was defined according to Sepsis-3 criteria; quick Sequential Organ Failure Assessment (qSOFA) was used only as a bedside screening tool. Statistical tests were selected according to variable type, distribution, and expected cell counts. **Results:** The cohort comprised 157 unique patients, with a median age of 71 years (interquartile range [IQR], 61–76). Current CRE colonization was documented in 79/154 patients with available colonization status (51.3%). Complete-case in-hospital mortality was higher in the carbapenem-resistant group (CRKP + CP-KP, n = 46) than in the non-carbapenem-resistant group (CSKP + ESBL, n = 111): 11/42 (26.2%) versus 5/108 (4.6%; Fisher exact odds ratio (OR) 7.31, 95% confidence interval (CI) 2.36–22.65; *p* < 0.001); overall complete-case mortality was 16/150 (10.7%). Multivariable logistic regression for carbapenem resistance (N = 150; five prespecified covariates; events per variable (EPV) = 9.0) identified age 65 years or older (adjusted odds ratio [aOR] 3.78, 95% CI 1.32–10.86), recent hospitalization within 30 days (aOR 2.56, 95% CI 1.16–5.63), and current colonization (aOR 2.96, 95% CI 1.24–7.05) as independent predictors. CRE-associated sepsis was excluded a priori because of definitional circularity with the case definition. Male sex showed a non-significant protective trend (aOR 0.50, 95% CI 0.22–1.12). CRE-associated sepsis showed a strong bivariate association with carbapenem resistance (OR 9.90, 95% CI 3.91–25.09; *p* < 0.001), and this association is reported descriptively because the variable was excluded from the multivariable model owing to definitional circularity. Model performance was acceptable, with area under the curve (AUC) 0.77, Hosmer–Lemeshow *p* = 0.95, and Nagelkerke R^2^ = 0.25. Of 99 molecularly characterized isolates, OXA-48-like was detected in 78 (78.8%), NDM in 71 (71.7%), KPC in 6 (6.1%), and NDM + OXA-48-like dual production in 54 (54.5%); VIM and IMP were uniformly negative. **Conclusions:** In this high-risk hospital cohort, carbapenem resistance in *K. pneumoniae* was associated with advanced age, recent healthcare exposure, current CRE colonization, and a pronounced unadjusted mortality signal. Interpretation of sepsis and mortality requires explicit separation of colonization from active infection and invasive disease. These findings support intensified CRE surveillance, source-specific clinical interpretation, rapid resistance detection, and risk-adapted empirical antimicrobial strategies in high-risk hospital settings.

## 1. Introduction

Carbapenem resistance in *K. pneumoniae* represents one of the most serious global public health threats, owing to its rapid dissemination, limited therapeutic options, and strong association with adverse clinical outcomes [1,2]. Over the past decade, the clinical significance of carbapenem-resistant *K. pneumoniae* (CRKP) has increased markedly, driven by antimicrobial selective pressure, nosocomial transmission, and the remarkable genetic adaptability of this pathogen [2]. As a prominent member of the ESKAPE group, *K. pneumoniae* possesses both intrinsic and acquired resistance mechanisms, resulting in substantial morbidity, mortality, and economic burden worldwide [3].

The widespread use of carbapenems for the treatment of severe Gram-negative infections has played a key role in the selection and expansion of resistant clones, particularly in tertiary hospitals and intensive care units [4,5,6]. Molecular epidemiological studies consistently demonstrate the predominance of carbapenemase enzymes such as KPC, NDM, and OXA-48-like variants, which significantly compromise available treatment strategies [7,8]. Moreover, CRKP strains frequently harbor additional resistance determinants, leading to reduced susceptibility to last-line agents, including colistin, tigecycline, and aminoglycosides [9,10].

Clinically, invasive CRKP infections are associated with worse outcomes than carbapenem-susceptible infections, including higher mortality, prolonged hospitalization, and increased risk of septic shock [11,12,13,14]. Bloodstream infections and pneumonia caused by CRKP have been reported to carry mortality rates exceeding 40% in several single-center cohort studies [12,13,14].

Well-established risk factors for CRKP acquisition include prolonged hospital stay, intensive care unit admission, invasive procedures, severe comorbidities, and prior exposure to broad-spectrum antibiotics [15,16,17]. Gastrointestinal colonization may represent an important reservoir for subsequent infection in high-risk patients, but it should not be interpreted as invasive disease in the absence of compatible clinical criteria; this distinction supports the importance of active surveillance and targeted infection-control measures, particularly in vulnerable populations such as neonates, older adults, transplant recipients, and post-COVID-19 patients [13,18,19,20,21,22].

Despite extensive research, important gaps remain in understanding CRKP epidemiology across different settings. Reliance on single-center studies limits generalizability, while variability in diagnostics, stewardship, and infection control leads to inconsistent findings. Single-center cohort investigations are therefore essential to clarify risk factors and improve prediction of clinical outcomes.

This retrospective single-center cohort study was designed to evaluate clinical, epidemiological, microbiological, and treatment-related factors associated with *K. pneumoniae* colonization and infection, with particular focus on carbapenem-resistant and carbapenemase-producing phenotypes.

By integrating clinical, epidemiological, microbiological, and treatment-related data, the study sought to (1) differentiate colonization-only status from non-invasive infection, bloodstream infection, ventilator-associated pneumonia (VAP) when reliably documented, device-associated infection, and CRE-associated sepsis; (2) evaluate antimicrobial resistance profiles, carbapenemase profiles, and MIC distributions; (3) identify independent predictors of carbapenem resistance using clinically selected multivariable modeling; and (4) support risk-adapted prevention, early detection, and antimicrobial-management strategies for patients at high risk of CRKP colonization or infection.

Such analyses may support targeted interventions, strengthen antimicrobial stewardship, and improve clinically appropriate interpretation of *K. pneumoniae* isolation in high-risk hospital populations.

## 2. Materials and Methods

### 2.1. Study Design and Population

This was a single-center retrospective observational cohort study conducted at Prof. Dr. Agrippa Ionescu Clinical Emergency Hospital, Bucharest, Romania, between January and July 2025. No patients from other institutions were included in the analytical cohort.

Eligible patients were identified from hospital admission records and microbiology laboratory databases using a standardized extraction protocol applied throughout the study period.

Patients were included when all of the following criteria were met: (1) adults aged 18 years or older; (2) at least one microbiologically confirmed *K. pneumoniae* isolate recovered during hospitalization from urine cultures, respiratory specimens (tracheal aspirates, bronchoalveolar lavage, or sputum), rectal surveillance swabs collected for CRE screening, blood cultures, device-associated specimens, or other clinically documented samples; (3) available clinical documentation for the index admission, including demographic data, healthcare exposure, comorbidities, antimicrobial exposure, infection status, treatment, and outcome; and (4) available antimicrobial susceptibility data for the corresponding isolate.

Patients were excluded in the following situations: incomplete species-level identification; isolates not confirmed as *K. pneumoniae*; polymicrobial specimens in which the clinical role of *K. pneumoniae* could not be reliably established; missing essential clinical or treatment data; unavailable antimicrobial susceptibility results; age <18 years; or discharge, transfer, or death before microbiological confirmation, when outcome and treatment data could not be reliably extracted.

All patients meeting the inclusion criteria were enrolled consecutively, ensuring representativeness of real-world clinical complexity and minimizing selection bias. No restrictions were applied regarding sex, ethnicity, comorbidity burden, prior healthcare exposure, invasive-device use, or prior antimicrobial history.

At hospital admission, patients provided general institutional consent for the use of anonymized clinical data for healthcare quality assessment and research purposes, according to local institutional policy. For this retrospective analysis, data were anonymized prior to analysis and no identifiable patient-level information was reported.

For each enrolled patient, demographic characteristics, prior healthcare exposures, comorbidities, procedural risk factors, colonization status, clinical syndrome, infection severity, antimicrobial resistance profiles, treatment adequacy, time to active therapy, and in-hospital outcome were extracted. This approach supported separate characterization of colonized and infected patients and allowed subsequent epidemiological, microbiological, and multivariable analyses.

This study included only *K. pneumoniae* isolates recovered from clinical specimens and/or CRE surveillance rectal swabs processed in the microbiology laboratory. No other bacterial species were included in the analysis.

All isolates were cultured under aerobic conditions at 37 °C using routinely employed culture media. Conventional media included Columbia agar with 5% sheep blood and MacConkey agar supplied by bioMérieux (Marcy-l’Étoile, France). Selective chromogenic media were used for resistance screening, including chromID^®^ ESBL agar and chromID^®^ CARBA SMART agar, both supplied by bioMérieux (Marcy-l’Étoile, France), according to the manufacturer’s instructions.

Bacterial identification and routine antimicrobial susceptibility testing were performed using the VITEK^®^ 2 XL system and VITEK^®^ 2 identification/AST cards (bioMérieux, Marcy-l’Étoile, France). Antimicrobial susceptibility results were interpreted according to EUCAST criteria applicable at the time of testing.

Carbapenemase production was investigated using NG-Test CARBA-5 (NG Biotech, Guipry, France) and PCR-based panels, as appropriate. For a subset of isolates, primarily those recovered from blood cultures, molecular confirmation was additionally performed using PCR-based panels, allowing detection of major carbapenemase genes. These molecular tests were applied selectively and not to all isolates.

Synergy testing between ceftazidime/avibactam and aztreonam was initially performed using a disk diffusion-based method, employing antibiotic disks supplied by Oxoid, Thermo Fisher Scientific (Basingstoke, UK), and Mueller-Hinton E agar supplied by bioMérieux (Marcy-l’Étoile, France).

Subsequently, this approach was replaced by ETEST^®^ aztreonam/avibactam gradient diffusion strips (bioMérieux, Marcy-l’Étoile, France). The use of ETEST^®^ aztreonam/avibactam allowed not only qualitative assessment of synergy, but also quantitative determination of minimum inhibitory concentrations (MICs), providing a more precise evaluation of antimicrobial activity.

Importantly, synergy testing and aztreonam/avibactam susceptibility testing were performed selectively, only for isolates resistant to ceftazidime/avibactam. These methods were not applied indiscriminately to all isolates.

Susceptibility to cefiderocol was assessed exclusively for ceftazidime/avibactam-resistant isolates, in parallel with synergy testing. Cefiderocol MICs were determined using UMIC Cefiderocol broth microdilution strips/panels (Bruker Daltonics GmbH & Co. KG, Bremen, Germany), in accordance with EUCAST recommendations. Cefiderocol was evaluated only as a single agent, and no in vitro synergy testing involving cefiderocol was performed.

All antimicrobial susceptibility testing results were interpreted according to EUCAST breakpoints and guidelines. Quality-control procedures were performed in accordance with routine laboratory standards.

Manufacturer names and locations were provided for the main diagnostic platforms, culture media, and antimicrobial susceptibility testing reagents. Catalog numbers were not consistently available in the retrospective laboratory documentation.

### 2.2. Data Sources and Clinical Variables

Data were extracted from electronic medical records and microbiology laboratory information systems, using a standardized case-report framework.

Demographic and epidemiological variables included source of admission (community, internal hospital units, external hospitals), recent hospitalization (within 30 days, 30 days to 3 months, none), ICU admission during current episode, and total length of hospital stay.

Comorbid conditions and baseline clinical status included obesity, diabetes mellitus, chronic pulmonary disease, chronic liver disease, autoimmune disease or immunosuppressive therapy, neoplastic disease under treatment or surveillance, and HIV status.

Procedural and device-related exposures included urinary catheterization, central venous catheterization, mechanical ventilation, prosthetic material, other invasive devices, and surgical procedures performed during admission.

Colonization variables included current CRE colonization, historical *K. pneumoniae* colonization within the preceding three months, and the clinical syndrome assigned to the index isolate. Admission colonization was defined a priori as the first CRE-positive specimen within 48 h of admission (pre-existing carriage), and hospital-onset colonization was defined as first documentation after 48 h of admission (possible nosocomial acquisition). Because admission-to-culture timing was not consistently available for all records, admission versus hospital-onset colonization could not be reliably separated in the main analysis, and current colonization was reported in aggregate.

Therapeutic variables included systemic antimicrobial exposure within the preceding three months, empirical treatment activity, active monotherapy, active combination therapy, time to initiation of active therapy (<24 h, 24–72 h, >72 h), and in-hospital mortality. When class-specific antimicrobial exposure was unavailable, prior exposure was analyzed as a binary 90-day variable and addressed as a limitation.

All variables were categorized, coded, and tabulated according to the definitions used in this single-center cohort.

### 2.3. Microbiological Identification and Resistance Profiling

All *K. pneumoniae* isolates were processed in an accredited clinical microbiology laboratory.

#### 2.3.1. Sample Types

Analyzed specimen types included urine cultures, respiratory secretions, rectal surveillance swabs collected for CRE screening, blood cultures, and device-associated specimens.

#### 2.3.2. Antimicrobial Susceptibility Testing

Antimicrobial susceptibility was assessed using automated systems, supplemented with confirmatory methods according to EUCAST Standard [23].The following antimicrobial classes were included: carbapenems (meropenem, imipenem/relebactam); aminoglycosides (amikacin); polymyxins (colistin); β-lactam/β-lactamase inhibitors (ceftazidime–avibactam, ceftolozane–tazobactam); siderophore cephalosporins (cefiderocol); tigecycline, fosfomycin, and trimethoprim–sulfamethoxazole.

Based on phenotypic susceptibility and the NG-Test CARBA-5 immunochromatographic assay (with PCR confirmation when available), each isolate was assigned hierarchically to one mutually exclusive group: (i) CP-KP—carbapenem non-susceptible and positive carbapenemase production (KPC, NDM, OXA-48-like, VIM or IMP); (ii) CRKP—carbapenem non-susceptible without confirmed carbapenemase (porin- or efflux-mediated resistance); (iii) ESBL—carbapenem-susceptible and phenotypically ESBL-positive; or (iv) CSKP—carbapenem-susceptible and ESBL-negative. MDR and XDR designations [18] are orthogonal descriptors, tabulated separately and not used as stratifying groups.

Antimicrobial susceptibility data were drawn from two parallel datasets: (i) the routine clinical antibiogram performed on all 157 isolates by the VITEK 2 platform, with cefiderocol and aztreonam/avibactam testing performed selectively in isolates with phenotypic ceftazidime/avibactam resistance; and (ii) an extended synergy panel performed on selected isolates (n = 54) with numerical MIC values for cefiderocol, aztreonam/avibactam, tigecycline, and eravacycline. The two datasets are reported separately because they reflect different testing populations and methodologies.

#### 2.3.3. MIC Distribution and Enzymatic Profiling

Minimum inhibitory concentration (MIC) values for cefiderocol, ceftazidime–avibactam, colistin, and meropenem were recorded and compared across carbapenemase types: KPC; NDM; OXA-48-like; NDM/OXA-48-like co-producers; and non-determinable enzyme profiles.

MIC clusters were analyzed to determine the relationship between enzymatic patterns and elevated resistance.

### 2.4. Definitions

- CRE colonization/carrier status was assigned to patients with positive rectal surveillance swabs or clinical specimens positive for carbapenem-resistant Enterobacterales. Rectal-swab positivity was interpreted as gastrointestinal colonization and not as invasive infection.
- Recent colonization was defined as documented carriage of *Klebsiella pneumoniae* within the previous three months (<3 months), based on clinical or active surveillance cultures.
- Sepsis was defined according to Sepsis-3 criteria as an acute Sequential Organ Failure Assessment (SOFA) score increase of at least 2 points in the context of documented or clinically suspected infection; quick Sequential Organ Failure Assessment (qSOFA) was used only as a bedside screening tool and was not considered a diagnostic criterion.
- CRE-associated sepsis required a Sepsis-3 episode, plus chart-based clinical adjudication of a CRE isolate as causative, with: (i) isolation within ±48 h of organ dysfunction from an anatomically compatible site; (ii) absence of a more plausible alternative pathogen; and (iii) clinical course consistent with anti-CRE therapy. Rectal-swab-only isolates were not classified as CRE-associated sepsis.

Each patient was assigned by chart review blinded to resistance phenotype to one of five mutually exclusive clinical-syndrome categories, applied hierarchically from most to least invasive: (1) bloodstream infection (BSI)—positive blood culture with Sepsis-3 organ dysfunction; (2) pneumonia/VAP—positive lower-respiratory specimen with new infiltrate and systemic features when reliably documented; (3) device-associated infection—clinically symptomatic infection attributable to a catheter, prosthetic, or urinary device; (4) non-invasive infection—localized infection without organ dysfunction (typically uncomplicated UTI); and (5) colonization only—rectal swab or clinical isolate without infection signs.

Active antimicrobial therapy was defined as administration of at least one systemic antimicrobial agent with documented in vitro activity against the index isolate according to EUCAST breakpoints, prescribed at a dose and route appropriate to the infection site, renal function, and severity of illness. Tigecycline monotherapy was not considered active therapy for bloodstream infection or pneumonia, and colistin was considered active only when loading and maintenance dosing were documented. Time zero (t_0_) was the collection time of the first clinical specimen yielding the index isolate; for patients first identified through surveillance swabs, time zero was reset to the first clinically relevant specimen. Time to active therapy was categorized as <24 h, 24–72 h, or >72 h; delayed active therapy was defined as initiation after >72 h.

### 2.5. Statistical Analysis

All analyses used IBM SPSS Statistics v27 with independent verification in Python (SciPy 1.13, statsmodels 0.14); a two-sided α = 0.05 was applied. Reported *p*-values are unadjusted; no formal correction for multiple testing was performed, given the descriptive nature of the cohort comparisons and the exploratory characterization of risk-factor associations.

#### 2.5.1. Descriptive Statistics

Continuous variables were assessed for normality by Shapiro–Wilk. Age (W = 0.91, *p* < 0.001) and length of stay (W = 0.14, *p* < 0.001) were non-normal; continuous variables are therefore reported as median (interquartile range). Categorical variables are reported as frequencies and percentages with non-missing denominators specified per table.

#### 2.5.2. Group Comparisons

The primary stratification used the four mutually exclusive phenotypic groups (Section 2.3.2); a prespecified secondary stratification compared CRKP + CP-KP versus CSKP + ESBL. Continuous variables were compared across the four phenotypic groups, using the Kruskal–Wallis H test. Categorical variables were compared with Pearson’s χ^2^ test when all expected counts were at least 5 and with Fisher exact test otherwise (Freeman–Halton extension with Monte-Carlo *p*-values for sparse r × c tables). Effect sizes are reported as odds ratios with Wald 95% confidence intervals (Haldane–Anscombe 0.5 correction for zero cells); risk ratios are reported alongside ORs for patient-level outcomes.

#### 2.5.3. Correlation Analysis

Pearson correlation was not used. Pairwise associations between binary clinical variables were quantified by the phi (φ) coefficient with 95% bootstrap confidence intervals (2000 resamples).

#### 2.5.4. Multivariable Logistic Regression

Prespecified covariates were selected on a priori clinical grounds: age 65 years or older, male sex, invasive devices, recent hospitalization within 30 days, and current CRE colonization. CRE-associated sepsis, although strongly associated with carbapenem-resistance status in bivariate analysis (Fisher exact OR 9.90, 95% CI 3.91–25.09, *p* < 0.001), was not included as a covariate in the multivariable model because the case-defining criterion for CRE-associated sepsis required a CRE isolate (Section 2.4), generating circularity with the binary outcome (CR/CP-KP vs. CSKP/ESBL). The bivariate association is reported descriptively in Table 1 and Section 3.3. ICU admission and antibiotic exposure within 3 months were not included as multivariable covariates given that a priori prioritization aimed at maintaining the events-per-variable ratio close to the conventional threshold of 10 (see Section 4.6, Limitations). Adjusted odds ratios (aORs) with 95% Wald confidence intervals are reported; model performance was assessed by the Hosmer–Lemeshow goodness-of-fit test, Nagelkerke R^2^, and the area under the ROC curve.

A prespecified second logistic model for in vitro aztreonam + ceftazidime/avibactam synergism was not estimated because the events-per-variable ratio was below the conventional threshold for stable estimation (16 synergy-positive isolates of 83 tested); descriptive associations between carbapenemase profile and synergism are reported in Section 3.4 instead.

#### 2.5.5. Handling of Missing Data

Outcome and exposure variables were complete for at least 88% of the cohort, one exception being in-hospital mortality, with approximately 4.5% (7 of 157) missing discharge documentation. Analyses used complete-case logic with operative denominators reported per table.

## 3. Results

### 3.1. Main Sample Description

In the canonical cohort of 157 patients, current CRE colonization was documented in 79 of 154 patients with a non-missing colonization flag (51.3%). Reliable separation of admission colonization (≤48 h post-admission) from hospital-onset colonization (>48 h post-admission) could not be performed because admission-to-culture timing was not consistently recorded in the administrative dataset (Section 4.6, Limitations); current colonization is therefore reported in aggregate. Historical colonization within the preceding three months is summarized in updated Table 1.

**Table 1:** Patient profiles and clinical and microbiological characteristics of the study cohort in the canonical N = 157 cohort. Phenotype distribution: CSKP (n = 93), ESBL-producing (n = 18), CRKP (n = 9), CP-KP (n = 37). *p*-values were obtained using chi-square tests or Fisher exact tests when expected counts were <5; age was compared by Kruskal–Wallis test.

Factor	Category	Total n (%)	CSKP (n = 93)	ESBL (n = 18)	CRKP (n = 9)	CP-KP (n = 37)	*p*-Value
Sex	Male	84 (53.5)	49 (52.7)	13 (72.2)	2 (22.2)	20 (54.1)	0.107
	Female	73 (46.5)	44 (47.3)	5 (27.8)	7 (77.8)	17 (45.9)	0.107
Age, years	Median (IQR)	71 (61–76)	68 (57–74)	72 (65–77)	72 (68–73)	75 (69–80)	0.007
Source of admission	Community	94 (60.6)	65 (70.7)	8 (44.4)	6 (66.7)	15 (41.7)	0.026
	Internal hospital	52 (33.5)	24 (26.1)	9 (50.0)	3 (33.3)	16 (44.4)	
	External hospital	9 (5.8)	3 (3.3)	1 (5.6)	0 (0.0)	5 (13.9)	
Recent hospitalization ≤ 30 days	Yes	51 (32.7)	20 (21.5)	7 (41.2)	5 (55.6)	19 (51.4)	0.003
	No	105 (67.3)	73 (78.5)	10 (58.8)	4 (44.4)	18 (48.6)	0.003
Prior antibiotic exposure < 3 months	Yes	60 (38.2)	27 (29.0)	10 (55.6)	2 (22.2)	21 (56.8)	0.007
	No	97 (61.8)	66 (71.0)	8 (44.4)	7 (77.8)	16 (43.2)	0.007
Current colonization	Yes	79 (51.3)	34 (37.0)	13 (76.5)	4 (44.4)	28 (77.8)	<0.001
	No	75 (48.7)	58 (63.0)	4 (23.5)	5 (55.6)	8 (22.2)	<0.001
Historical colonization < 3 months	Yes	38 (24.5)	14 (15.1)	6 (35.3)	2 (22.2)	16 (44.4)	0.004
	No	117 (75.5)	79 (84.9)	11 (64.7)	7 (77.8)	20 (55.6)	0.004
ICU admission	Yes	72 (47.4)	34 (37.8)	9 (52.9)	4 (44.4)	25 (69.4)	0.014
	No	80 (52.6)	56 (62.2)	8 (47.1)	5 (55.6)	11 (30.6)	0.014
Invasive devices	Yes	79 (50.3)	38 (40.9)	11 (61.1)	5 (55.6)	25 (67.6)	0.034
	No	78 (49.7)	55 (59.1)	7 (38.9)	4 (44.4)	12 (32.4)	0.034
CRE-associated sepsis	Yes	28 (18.4)	6 (6.6)	2 (12.5)	2 (22.2)	18 (50.0)	<0.001
	No	124 (81.6)	85 (93.4)	14 (87.5)	7 (77.8)	18 (50.0)	<0.001
In-hospital mortality	Yes	16 (10.7)	3 (3.3)	2 (11.8)	0 (0.0)	11 (33.3)	<0.001
	No	134 (89.3)	88 (96.7)	15 (88.2)	9 (100.0)	22 (66.7)	<0.001

CSKP—carbapenem-susceptible *Klebsiella pneumoniae*; CRKP—carbapenem-resistant *K. pneumoniae*; ESBL—extended-spectrum β-lactamase; CP-KP—carbapenemase-producing *Klebsiella pneumoniae*; ICU—intensive care unit.

#### Clinical Syndrome Distribution

In the canonical cohort of 157 patients, clinical syndromes were distributed as follows: bloodstream infection, nine (5.7%); device-associated infection, 20 (12.7%); non-invasive infection, 106 (67.5%); and colonization-only, 20 (12.7%). Two patients had insufficient data for assignment. Pneumonia/VAP could not be reliably assessed as a separate stratum because the source-specimen field did not consistently distinguish lower-respiratory infection from colonization or non-diagnostic respiratory isolation. Bloodstream and device-associated syndromes were disproportionately represented in the CR/CP-KP groups, whereas colonization-only and non-invasive infection dominated in the CSKP and ESBL groups.

### 3.2. Antibiotic Susceptibility Profiles of Klebsiella pneumoniae Isolates

The antimicrobial susceptibility profile was heterogeneous across the cohort, reflecting the mixed distribution of susceptible, ESBL-producing, carbapenem-resistant, and carbapenemase-producing isolates. Table 2 summarizes the overall susceptibility rates for the tested antimicrobial agents, using the available denominator for each drug. The complete antimicrobial susceptibility testing result are provided in Appendix A.

### 3.3. Clinical, Epidemiological, and Microbiological Characteristics of the Study Population

The secondary comparison between carbapenem-resistant and non-carbapenem-resistant groups identified several clinical and epidemiological factors associated with resistance. Table 3 presents the corresponding odds ratios, risk ratios, confidence intervals, and *p*-values.

### 3.4. Distribution of MIC Values and Enzymatic Profiles

Cefiderocol MIC values ranged from 0.125 to 16 mg/L, with a modal value of 1 mg/L; in the consolidated cohort, 43 of 54 isolates (79.6%) were categorized as susceptible by EUCAST breakpoints. A resistant tail was present at MIC 4–16 mg/L (Table 4). Among the 46 isolates with both numerical MIC and complete molecular profile (Table 4), elevated cefiderocol MIC of 4 mg/L or higher was observed in eight of 41 NDM-containing isolates versus one of five NDM-negative isolates (Fisher exact OR 0.97, 95% CI 0.10 to 9.90, *p* = 1.00). The comparison is statistically uninformative owing to the extreme group imbalance (only five NDM-negative isolates with MIC data) and is reported descriptively rather than inferentially. Patient-level clinical outcomes for the elevated-MIC subset were unavailable in this dataset; the clinical implications of the resistance tail are therefore presented as in vitro observations rather than evidence of clinical effectiveness.

Among the 41 *K. pneumoniae* isolates tested for aztreonam/avibactam, all were categorized as susceptible relative to the PK/PD target of 4 mg/L or lower; numerical MIC values were available for 39 isolates and showed a narrow dispersion preserved across all carbapenemase profiles, including NDM + OXA-48-like dual producers. Because testing was performed selectively only on ceftazidime/avibactam-resistant isolates, this finding is reported as a descriptive in vitro observation and not as evidence of clinical effectiveness in this cohort.

### 3.5. Association Between Clinical Factors, Colonization, Infection Severity, and Antimicrobial Exposure

Exploratory pairwise associations were assessed to characterize relationships between clinical exposure variables, colonization, sepsis, and antimicrobial exposure. Table 5 summarizes these binary associations using the phi coefficient with bootstrap confidence intervals.

#### Carbapenemase Gene Distribution

Molecular characterization was performed on 104 *K. pneumoniae* isolates, of which 99 yielded a valid carbapenemase profile and five had no detectable carbapenemase gene, despite carbapenem non-susceptibility. Among the 99 with valid profiles, OXA-48-like was detected in 78 (78.8%), NDM in 71 (71.7%), KPC in six (6.1%), and ESBL co-production in 33 (33.3%). VIM and IMP were tested in all isolates and uniformly negative. Co-production of NDM and OXA-48-like was the modal profile (54 isolates), followed by OXA-48-like alone (24), NDM alone (15), and KPC alone or in combination (six). Of the 104 isolates, 50 underwent independent sequencing confirmation at the Cantacuzino National Institute, with 100% concordance for the principal carbapenemase calls. Patient-level linkage between the molecular dataset and the canonical clinical cohort was complete for only 23 of 157 patients (see Section 4.6 limitations).

### 3.6. Multivariable Analysis of Factors Associated with Carbapenem Resistance

A multivariable logistic regression analysis evaluated clinical and demographic factors associated with carbapenem resistance (Table 6). The model was constructed to estimate adjusted associations while accounting for prespecified healthcare exposure, colonization status, and demographic covariates.

### 3.7. Mortality Stratified by Clinical Syndrome and Phenotypic Group

In the canonical N = 157 cohort, in-hospital mortality was reconciled across the records: 16 confirmed deaths, 134 confirmed survivors, and seven with unresolved discharge status; the complete-case denominator is N = 150. Overall complete-case in-hospital mortality was 16/150 (10.7%), higher than the 7.0% figure derived from the original unreconciled N = 143 denominator. Two-group comparison of carbapenem-resistant (CRKP + CP-KP) versus non-carbapenem-resistant (CSKP + ESBL) groups yielded an unadjusted odds ratio for in-hospital mortality of 7.31 (95% CI 2.36–22.65; Fisher exact *p* < 0.001), with 11/42 deaths in the CR/CP-KP group and 5/108 in the CSKP/ESBL group. When further stratified by clinical syndrome, mortality concentrated in CP-KP patients with invasive syndromes (BSI or device-associated), whereas colonization-only patients had near-zero mortality regardless of phenotype. The pneumonia/VAP stratum could not be assessed because the source-specimen field did not distinguish lower-respiratory infection from colonization or non-diagnostic respiratory isolation (Section 4.6). The multivariable logistic regression for carbapenem resistance (Table 6; five prespecified covariates, complete-case N = 150, EPV = 9.0) yielded adjusted odds ratios of 3.78 (95% CI 1.32–10.86, *p* = 0.013) for age 65 years or older, 2.56 (95% CI 1.16–5.63, *p* = 0.020) for recent hospitalization within 30 days, and 2.96 (95% CI 1.24–7.05, *p* = 0.015) for current colonization; male sex showed a non-significant protective trend (aOR 0.50, 95% CI 0.22–1.12, *p* = 0.093). CRE-associated sepsis was excluded from the multivariable model a priori owing to circularity with the case definition; the strong bivariate association (Fisher exact OR 9.90, 95% CI 3.91–25.09, *p* < 0.001) is reported descriptively. The model demonstrated acceptable discrimination (AUC 0.77), well-calibrated fit (Hosmer–Lemeshow *p* = 0.95), and modest explanatory power (Nagelkerke R^2^ = 0.25).

## 4. Discussion

Antimicrobial resistance among *K. pneumoniae* remains a critical global threat, driven by the rapid emergence and dissemination of carbapenem-resistant Enterobacterales. Similar to trends reported internationally [21,22,24], our single-center cohort depicts a clinically complex patient population characterized by high comorbidity burden, significant healthcare exposure, and extensive antimicrobial selection pressure, conditions that facilitate the persistence and spread of carbapenem-resistant *K. pneumoniae*. Resistance in our cohort was better characterized using a mechanistic classification (CSKP, CRKP, ESBL-producing, and CP-KP), while the clinical interpretation of outcomes required explicit differentiation between colonization, non-invasive infection, invasive infection, and CRE-associated sepsis.

### 4.1. CRKP Epidemiology and Carriage Dynamics

The epidemiology of carbapenem-resistant *K. pneumoniae* in our cohort reflects the increasingly complex and high-pressure hospital environments in which these pathogens emerge and persist. Rather than representing sporadic events, CRKP cases occurred within a population characterized by substantial healthcare exposure, frequent invasive interventions, and a high baseline burden of antimicrobial resistance risk factors, consistent with observations from tertiary-care hospitals in endemic regions [25,26,27].

In the overall cohort, current CRE colonization was documented in 79 of 154 patients with available colonization status (51.3%), indicating a substantial *K. pneumoniae* carriage reservoir at this single tertiary-care center. Although the temporal distinction between admission colonization and hospital-onset colonization could not be reliably operationalized on the available administrative data (Section 4.6), the magnitude of carriage is consistent with regional surveillance from Central and Eastern European tertiary-care settings [28,29,30,31,32,33,34] and reflects both pre-existing community-or-prior-healthcare-acquired colonization and probable in-hospital transmission pressure.

Gastrointestinal colonization represents a reservoir for CRKP transmission and may precede subsequent infection in high-risk patients, but it should not be interpreted as invasive disease in the absence of compatible clinical criteria. The high prevalence of current colonization observed in our cohort exceeds baseline rates reported in national point prevalence surveys and reflects the accumulation of epidemiological risk within a clinically complex hospital population [25,31]. Sustained transmission pressure is likely facilitated by prolonged hospitalization, frequent patient transfers, and concentration of vulnerable patients within intensive and units providing highly acute care [26,35].

Colonization was associated with resistant phenotypes and should be interpreted as an epidemiological reservoir and risk marker rather than as evidence by itself of invasive disease. Patients with CRKP and carbapenemase-producing *K. pneumoniae* exhibited significantly higher rates of both current and recent colonization compared with those harboring carbapenem-susceptible or ESBL-producing isolates. This finding supports the concept that colonization identifies patients at higher epidemiological risk, particularly when repeated healthcare interventions and antimicrobial pressure are present [30,33,36].

### 4.2. Carbapenemase Profiles and Implications for Antimicrobial Resistance

The distribution of carbapenemase types observed in our cohort reflects an advanced stage of antimicrobial resistance evolution, increasingly reported across hospital networks worldwide and characterized by the expansion of metallo-β-lactamase (MBL)-producing *K. pneumoniae*, particularly New Delhi metallo-β-lactamase (NDM), alongside the continued circulation of OXA-48-like enzymes [37,38,39]. Rather than being driven by a single dominant mechanism, carbapenem resistance in our setting was defined by heterogeneous and frequently overlapping enzymatic profiles, highlighting the growing complexity of CRKP epidemiology.

Among the 99 molecularly characterized isolates with a valid carbapenemase profile (Section 3.5 below), OXA-48-like was detected in 78 (78.8%), NDM in 71 (71.7%), KPC in six (6.1%), and the NDM + OXA-48-like dual-producer profile in 54 (54.5%); VIM and IMP were uniformly negative. NDM and OXA-48-like therefore represent the predominant resistance mechanisms in our setting. This pattern is consistent with reports from Southern and Eastern Europe, the Middle East, and Asia [40,41,42,43]. Dual-producer strains are of particular concern owing to enhanced horizontal gene transfer and broad resistance phenotypes [44,45].

From a microbiological perspective, the presence of NDM in the molecularly characterized subset was associated with elevated MIC values across most beta-lactam agents, as quantified in Table 4 (Section 3.4). The odds of an elevated cefiderocol MIC of 4 mg/L or higher among NDM-containing isolates were numerically similar to those in non-NDM isolates in the small available subset (OR 0.97, see Section 3.4) and should be interpreted descriptively. This observation aligns with intrinsic biochemical properties of MBLs, which efficiently hydrolyze carbapenems and are not inhibited by avibactam, vaborbactam or relebactam [37,46]. Consequently, NDM-producing isolates—particularly those co-harboring OXA-48-like—frequently exhibited extensively drug-resistant phenotypes [41,47].

Although mechanistic characterization was not performed in the present cohort, the observed pattern of elevated cefiderocol MIC in NDM-containing isolates is consistent with mechanisms described in the literature, including siderophore-receptor alterations and porin loss in metallo-β-lactamase-producing strains [42,48,49,50]. These findings reinforce the importance of monitoring last-line agents in NDM-prevalent settings.

### 4.3. Clinical Factors Associated with CRE-Associated Sepsis and Invasive Infection

The transition from CRKP colonization to invasive infection, when it occurs, is a multifactorial process shaped by healthcare exposure, procedural risk factors, host vulnerability, and microbial resistance profiles [3,5,15]. In our single-center cohort, the convergence of intensive care exposure, prior colonization, and invasive interventions created a high-risk clinical environment associated with both persistent carriage and clinically adjudicated CRE-associated sepsis [10,22,27].

ICU admission was more frequent among patients with resistant phenotypes in bivariate analyses and should be interpreted as a marker of critical illness, healthcare exposure, invasive procedures, and antimicrobial pressure. This interpretation is consistent with international cohorts in which critically ill patients experience disproportionate rates of CRKP colonization and infection [7,13,28,32,51]. In our study, ICU admission was not retained as an independent predictor in the final multivariable model, which prioritized covariates according to clinical relevance and events-per-variable constraints [19,25,52].

Invasive devices were more frequently observed among patients with resistant phenotypes and were associated with CRE-associated sepsis in exploratory binary-variable analyses [9,20,53]. They remained clinically relevant as markers of barrier disruption, biofilm risk, and healthcare exposure, although they were not independent predictors in the final multivariable model. Their role should therefore be interpreted clinically rather than as an adjusted independent effect [14,26,38].

Prior antibiotic exposure, while biologically plausible as a driver of colonization and resistance selection, showed only a modest direct association with CRE sepsis [11,24,33]. This suggests that antimicrobial pressure alone may be insufficient to precipitate invasive disease in the absence of additional clinical risk factors, particularly invasive devices and critical illness [17,40,54].

Overall, CRE-associated sepsis occurred within a high-risk clinical network characterized by current CRE colonization, recent healthcare exposure, invasive devices, ICU-level care, and antimicrobial selective pressure, rather than as a direct consequence of colonization alone [6,28,39,44,48].

### 4.4. Treatment Adequacy, Delays, and Mortality

In-hospital mortality in the canonical N = 157 cohort was 16/150 (10.7%) with complete-case ascertainment, against the 7.0% previously reported on the unreconciled denominator of 143. Although modest in absolute terms, the unadjusted odds ratio for carbapenem-resistant versus non-carbapenem-resistant *K. pneumoniae* was 7.31 (95% CI 2.36–22.65, *p* < 0.001), indicating a strong mortality signal that was previously obscured by the aggregate denominator. As detailed in Section 3.7, the syndrome composition of the cohort—dominated by non-invasive infection and colonization-only—accounts for the lower aggregate rate compared with the 30–60% commonly reported in CRKP bloodstream-infection cohorts [6,7,9,27,55,56].

First, the study population included both colonized and infected patients, with a predominance of non-invasive infections. Bloodstream infections accounted for a limited proportion of cases, whereas invasive disease was more frequently related to urinary tract, device-associated, or localized infections, which are generally associated with more favorable outcomes [1,3,12,20,57,58]. This distribution likely attenuated overall mortality, compared with cohorts restricted to CRKP bacteremia or ventilator-associated pneumonia [27,55].

Second, active surveillance practices and early microbiological identification may have facilitated earlier recognition of colonization or infection, increasing clinical vigilance and enabling targeted interventions before clinical deterioration or invasive infection occurred [8,18,24,51]. Although rectal screening was not uniformly applied to all admissions, its use in high-risk patients likely contributed to earlier detection of CRE carriage and informed infection control and therapeutic decision-making [15,32,59].

The mismatch between empirical therapy and resistance profiles reflects the complexity of treating infections caused by MBL-producing CRKP, particularly NDM and NDM/OXA-48-like strains [37,38,39,40,41]. In such settings, commonly used empirical regimens, including carbapenems, piperacillin–tazobactam, and aminoglycosides, frequently lack activity [19,29,35]. The need to await phenotypic susceptibility results or carbapenemase identification may delay escalation to susceptibility-guided agents, including aztreonam/avibactam or cefiderocol when supported by in vitro testing and clinical context, underscoring the diagnostic gap between culture acquisition and actionable results [25,42,43,44,52].

Despite delays in active therapy, aggregate mortality remained moderate, probably reflecting the mixed case composition of the cohort and the inclusion of colonization-only and non-invasive infection cases. This may also reflect supportive care measures, source control interventions, and subsequent optimization of antimicrobial therapy once susceptibility data became available [5,13,21,53]. In addition, multidisciplinary management and the selective use of rescue or combination regimens in advanced resistance phenotypes may have mitigated the clinical impact of initial non-active therapy [45,46,47,54].

Nevertheless, the high frequency of delayed or inadequate empirical treatment highlights a critical need for strengthened antimicrobial stewardship strategies. Optimization of empirical therapy in high-risk units, wider implementation of rapid carbapenemase detection assays, and closer integration between microbiology laboratories and clinical teams are essential to reduce time to active treatment [14,22,30,36,48,49,50]. In settings with a high prevalence of MBL-producing strains, institutional treatment algorithms should be regularly updated to reflect local epidemiology and emerging resistance patterns [38,60,61].

### 4.5. Comparison with National and Regional Trends

The epidemiological patterns observed in our cohort are broadly consistent with trends reported by national and regional antimicrobial resistance surveillance programs across Europe, as well as with data from selected countries in Asia and the Middle East [25,28,29,33,55]. Recent European surveillance reports have documented a sustained increase in both colonization and infection caused by carbapenem-resistant Enterobacterales, with *Klebsiella pneumoniae* remaining the predominant species involved. Similar to our findings, countries in Central and Eastern Europe, including Romania, Bulgaria, Serbia, and Greece, have reported a gradual but persistent rise in CRKP prevalence, largely driven by healthcare-associated transmission, prolonged hospitalization, and repeated antimicrobial exposure [28,29,34].

Importantly, the enzymatic profiles identified in our cohort reflect a broader regional transition from OXA-48-like predominance toward an increasing contribution of metallo-β-lactamase-producing strains, particularly NDM. This shift has been increasingly described in surveillance data and observational studies published between 2018 and 2024, in which NDM-producing *K. pneumoniae* have progressively expanded alongside or replaced OXA-48-like and KPC-producing lineages in several endemic settings [20,21,22,37]. In our cohort, NDM and NDM/OXA-48-like carbapenemases accounted for a substantial proportion of carbapenem-resistant isolates, consistent with this evolving resistance landscape.

Evidence from large-scale genomic and epidemiological studies conducted in China, India, the Persian Gulf region, North Africa, and Turkey has similarly highlighted the growing prevalence of NDM and dual NDM/OXA-48-like producers, which are frequently associated with plasmid-mediated dissemination, high antimicrobial selective pressure, and the spread of successful high-risk clones such as ST11 and ST147 [19,22,37,62]. Although molecular typing was not systematically performed in our study, the phenotypic resistance patterns and carbapenemase profiles observed align with these internationally reported trends.

The convergence of epidemiological features across regions underscores the increasingly interconnected nature of CRKP dissemination. In Asian settings, the dominance of NDM-producing strains has been closely linked to intensive care exposure, extensive device use, and widespread carbapenem consumption [22,37]. Comparable patterns have been described in Middle Eastern and North African countries, where dual-carbapenemase producers are emerging with increasing frequency and are often associated with limited therapeutic options and reduced susceptibility to last-line agents such as colistin [33,37,63]. These observations parallel the resistance phenotypes documented in our cohort, particularly among MDR and XDR isolates.

At the national level, the prevalence and resistance profiles observed in our study are consistent with data reported in recent intra-hospital surveys, point prevalence studies, and single-center observational cohorts from European healthcare systems. In Romania and neighboring countries, surveillance data suggest a transition from predominantly OXA-48-like producers before 2020 toward more heterogeneous carbapenemase distributions dominated by NDM and hybrid producers after 2021, a pattern that closely mirrors our findings. Furthermore, the fact that approximately one-third of patients in our cohort were admitted from external healthcare facilities supports the concept of regional circulation and inter-facility transmission of CRE strains.

Taken together, the alignment between our local data and national and international surveillance trends indicates that the epidemiology of CRKP in our setting reflects a broader global shift toward increasing resistance complexity. The growing predominance of MBL-associated resistance, particularly involving NDM and NDM/OXA-48-like co-producers, highlights the urgent need for coordinated infection prevention strategies, enhanced molecular surveillance, and harmonized antimicrobial stewardship efforts at both national and cross-border levels to limit further dissemination within interconnected healthcare networks.

### 4.6. Limitations

Several limitations of this study should be acknowledged. First, the sample size was relatively modest, which may have limited the statistical power of multivariable analyses and reduced the generalizability of the findings to other healthcare settings or broader populations. Although the cohort was clinically heterogeneous and representative of a high-risk hospital population, the single-center nature of the present analysis restricts extrapolation to other institutions with different epidemiological pressures or infection-control practices.

Second, the retrospective observational design precludes the establishment of causal relationships between clinical exposures and outcomes. Data were extracted from routinely collected clinical and microbiological records, which may have resulted in incomplete documentation of certain variables, including the exact timing of invasive device placement or removal, the duration of antibiotic exposure, and subtle or transient colonization episodes. As a result, misclassification or underestimation of some risk factors cannot be excluded.

Third, colonization screening was not performed systematically for all patients at admission or throughout hospitalization. Screening was largely driven by clinical indication or local surveillance protocols, which may have led to undetected early or asymptomatic colonization. Consequently, some cases classified as newly acquired colonization may in fact have represented previously unrecognized colonization, potentially biasing estimates of colonization dynamics.

Fourth, molecular characterization of carbapenemase genes was available only for a subset of isolates. While phenotypic carbapenemase detection was performed for all relevant isolates and provided reliable enzymatic classification, the limited availability of molecular data precluded detailed assessment of clonal relatedness, plasmid architecture, or transmission pathways. This restriction limits insight into the genomic epidemiology underlying the observed resistance patterns.

Fifth, prior antimicrobial exposure was captured as a binary indicator of any systemic antimicrobial therapy within the three months preceding admission, without resolution at the level of antimicrobial class, individual agent, or duration. Pharmacy dispensing records with the required granularity were available only for the index admission. Consequently, the present study cannot resolve the differential selection pressure exerted by carbapenems, third- and fourth-generation cephalosporins, fluoroquinolones, piperacillin/tazobactam, aminoglycosides, colistin, or oral beta-lactams, all of which have agent-specific associations with CRKP selection [17,40,54]; the binary three-month exposure variable should therefore be interpreted as a coarse proxy for cumulative antimicrobial pressure.

Finally, analyses of treatment adequacy, delays to active therapy, and clinical outcomes were conducted without adjustment for certain important confounders, such as validated severity-of-illness scores, timing and effectiveness of source control, or the presence of concurrent infections. These factors may have influenced both therapeutic decision-making and patient outcomes and could partially account for the observed associations between delayed or non-active therapy and mortality.

Despite these limitations, the study provides valuable real-world data on the epidemiology, resistance mechanisms, and clinical impact of carbapenem-resistant *K. pneumoniae* in a high-risk hospital population. The integration of clinical, microbiological, and therapeutic variables offers a clinically relevant perspective on colonization dynamics, invasive infection, and antimicrobial-management challenges in a high-risk tertiary-care setting.

In addition, the multivariable logistic regression for carbapenem resistance was constrained by the events-per-variable ratio: with 45 events and five prespecified covariates, the EPV was 9.0, marginally below the conventional threshold of 10. Adjusted estimates are therefore reported with confidence intervals that reflect this limited precision and should be interpreted as hypothesis-generating rather than confirmatory; external validation in larger multicenter cohorts is needed. CRE-associated sepsis, the strongest bivariate predictor of carbapenem resistance, was excluded a priori from the multivariable model because its case definition required a CRE isolate, generating circularity with the outcome; the bivariate association is reported descriptively in Section 3.3 and Table 1.

### 4.7. Implications for Clinical Practice

The present findings have several important implications for clinical practice, particularly in hospital settings providing highly acute care.

First, the predominance of carbapenem-resistant *K. pneumoniae*, including a substantial proportion of carbapenemase-producing isolates, highlights the importance of early identification of resistance mechanisms. In this cohort, phenotypic carbapenemase detection was routinely available, while molecular confirmation was performed selectively. These results support the clinical value of integrating rapid carbapenemase detection strategies, especially in intensive care units and among patients with recent hospitalization or known colonization, to facilitate earlier optimization of antimicrobial therapy.

Second, the association between ICU stay, invasive-device use, and CRE-associated sepsis in bivariate analyses reinforces the critical role of device stewardship in preventing severe infections. Minimizing unnecessary catheterization, ensuring timely removal of invasive devices, and maintaining strict adherence to aseptic insertion and maintenance protocols may reduce the risk of invasive infection among colonized high-risk patients.

Third, the high frequency of non-active empirical therapy and delays exceeding 72 h before initiation of active treatment underscores important gaps in empirical management strategies for high-risk patients. These findings suggest that empirical treatment algorithms in settings with a high burden of CRKP should incorporate individual risk stratification based on prior colonization, recent healthcare exposure, ICU admission, and local resistance patterns, rather than relying solely on standard broad-spectrum regimens.

### 4.8. Policy and Public Health Implications

The findings of this study also carry broader implications for infection prevention policy and public health planning.

The high prevalence of current and recent colonization observed in this cohort highlights the importance of structured CRE surveillance programs, particularly for patients admitted to ICUs or transferred from other healthcare facilities. Targeted admission screening of high-risk patients may facilitate earlier identification of carriers and reduce silent transmission within hospital wards.

Strengthening antimicrobial stewardship programs remains essential. The association between recent antimicrobial exposure and carbapenem resistance observed in this study supports policies aimed at optimizing antimicrobial prescribing, limiting unnecessary use of broad-spectrum agents, and reinforcing audit-and-feedback mechanisms, especially in high-risk units.

At an institutional and regional level, the results support the integration of phenotypic and, where feasible, molecular surveillance data into routine reporting systems. Such integration would enhance situational awareness of local resistance patterns, support timely infection-control interventions, and inform procurement and prioritization of last-line antimicrobial agents.

### 4.9. Directions for Future Research

Future research should target prospective study designs with systematic rectal carriage screening at admission and during hospitalization to better characterize colonization dynamics and clarify temporal relationships between colonization and infection.

Expanded molecular epidemiology, including whole-genome sequencing, would enable deeper insights into clonal dissemination, plasmid-mediated resistance mechanisms, and transmission pathways within and between healthcare facilities. Such data would complement the phenotypic findings of the present study and support targeted infection-control strategies.

In addition, further clinical studies are needed to evaluate the real-world effectiveness of combination and synergy-guided therapies in settings with a high prevalence of carbapenemase-producing and XDR organisms. Longitudinal follow-up of colonized patients would also help clarify long-term outcomes, recurrence rates, and the risk of inter-facility transmission.

## 5. Conclusions

Carbapenem-resistant *K. pneumoniae* was associated with CRE-associated sepsis in bivariate analysis (OR 9.90, *p* < 0.001), while advanced age, recent healthcare contact, and current colonization remained independently associated with carbapenem resistance in multivariable analysis. The strong unadjusted CR/CP-KP mortality signal (OR 7.31, *p* < 0.001) should be interpreted in relation to cohort composition, which included colonization-only cases and non-invasive infections.

Overall, these findings reinforce the need for integrated infection-prevention strategies, enhanced CRE surveillance, source-specific clinical interpretation, and risk-adapted antimicrobial stewardship in environments providing highly acute care. By aligning clinical decision-making with local epidemiology and resistance patterns, healthcare systems may improve outcomes and mitigate the ongoing impact of carbapenem-resistant *K. pneumoniae*.

## Figures and Tables

**Table 2:** Antimicrobial susceptibility rates of *K. pneumoniae* isolates in the canonical N = 157 cohort (overall susceptibility, all phenotypes combined; per-drug N reflects the subset of isolates with valid SIR results).

Antibiotic	N Tested	S, n (%)	I, n (%)	R, n (%)
Meropenem	114	89 (78.1%)	2 (1.8%)	23 (20.2%)
Cefiderocol	10	6 (60.0%)	0 (0.0%)	4 (40.0%)
Ceftazidime/avibactam	40	31 (77.5%)	0 (0.0%)	9 (22.5%)
Aztreonam	12	1 (8.3%)	0 (0.0%)	11 (91.7%)
Imipenem/relebactam	45	25 (55.6%)	0 (0.0%)	20 (44.4%)
Amikacin	102	91 (89.2%)	0 (0.0%)	11 (10.8%)
Colistin	51	31 (60.8%)	1 (2.0%)	19 (37.3%)
TMP-SMX	109	59 (54.1%)	0 (0.0%)	50 (45.9%)
Fosfomycin	7	1 (14.3%)	1 (14.3%)	5 (71.4%)
Tigecycline ^†^	47	39 (83.0%)	0 (0.0%)	8 (17.0%)
Eravacycline	1	1 (100.0%)	0 (0.0%)	0 (0.0%)

^†^ Tigecycline susceptibility was interpreted according to applicable criteria at the time of testing.

**Table 3:** Comparison of clinical and microbiological characteristics between carbapenem-resistant (CR/CP-KP, n = 46) and non–carbapenem-resistant (CSKP/ESBL, n = 111) *K. pneumoniae* groups in the canonical N = 157 cohort. Odds ratios with 95% Wald CI (Haldane–Anscombe correction when any zero cell); risk ratios with 95% Wald CI; *p*-values from Fisher exact test.

Factor	CR/CP-KP (n = 46)	CSKP/ESBL (n = 111)	OR (95% CI)	RR (95% CI)	*p*-Value
Male sex	22 (47.8)	62 (55.9)	0.72 (0.36–1.44)	0.86 (0.61–1.21)	0.384
Age 65 years or older	40 (87.0)	69 (63.9)	3.77 (1.47–9.68)	1.36 (1.14–1.63)	0.004
Hospital-origin admission (internal/external)	24 (53.3)	37 (33.6)	2.25 (1.11–4.57)	1.59 (1.09–2.32)	0.030
Recent hospitalization within 30 days	24 (52.2)	27 (24.5)	3.35 (1.63–6.91)	2.13 (1.38–3.26)	0.001
Prior antibiotic exposure < 3 months	23 (50.0)	37 (33.3)	2.00 (0.99–4.03)	1.50 (1.01–2.22)	0.071
Current colonization	32 (71.1)	47 (43.1)	3.25 (1.54–6.86)	1.65 (1.24–2.19)	0.002
Historical colonization < 3 months	18 (40.0)	20 (18.2)	3.00 (1.39–6.47)	2.20 (1.29–3.75)	0.007
ICU admission	29 (64.4)	43 (40.2)	2.70 (1.31–5.56)	1.60 (1.17–2.20)	0.008
Invasive devices	30 (65.2)	49 (44.1)	2.37 (1.16–4.84)	1.48 (1.10–1.99)	0.022
CRE-associated sepsis	20 (44.4)	8 (7.5)	9.90 (3.91–25.09)	5.94 (2.83–12.49)	<0.001
In-hospital mortality	11 (26.2)	5 (4.6)	7.31 (2.36–22.65)	5.66 (2.09–15.30)	<0.001

**Table 4:** MIC distribution of cefiderocol, aztreonam/avibactam, tigecycline, and eravacycline on the canonical N = 157 cohort. N (MIC) represents isolates with numerical MIC measurements; N (SIR) represents isolates with categorical susceptibility interpretation. These denominators may differ when categorical susceptibility was available without a numerical MIC value.

Antibiotic	N (MIC)	Range (mg/L)	Modal MIC (mg/L)	S, n (%)	I, n (%)	R, n (%)	N (SIR)
Cefiderocol	54	0.125–16	1	43 (79.6%)	0 (0.0%)	11 (20.4%)	54
Aztreonam/avibactam	39	0.094–0.75	0.25	41 (100.0%)	0 (0.0%)	0 (0.0%)	41
Tigecycline	10	0.5–1	0.5	12 (100.0%)	0 (0.0%)	0 (0.0%)	12
Eravacycline	1	0.38–0.38	0.38	1 (100.0%)	0 (0.0%)	0 (0.0%)	1

**Table 5:** Associations between clinical factors, colonization, infection severity, and antimicrobial exposure in the canonical N = 157 cohort. Phi coefficient (φ) for binary–binary pairs with 95% bootstrap CI (2000 resamples); *p*-values from chi-square (or Fisher exact when expected counts < 5).

Variable Pair	Coefficient	r/φ/ρ	95% CI (Bootstrap)	*p*-Value
ICU stay—Recent hospitalization	φ	0.135	[−0.031, 0.291]	0.096
Invasive devices—Sepsis	φ	0.204	[0.053, 0.348]	0.012
ICU—Sepsis	φ	0.236	[0.087, 0.386]	0.004
Invasive devices—CRE colonization	φ	0.351	[0.207, 0.493]	<0.001
Recent antibiotic—CRE colonization	φ	0.314	[0.166, 0.460]	<0.001
Recent antibiotic—Recent Hospitalization	φ	0.443	[0.295, 0.591]	<0.001
Invasive devices—Recent hospitalization	φ	0.141	[−0.011, 0.287]	0.077
ICU—Antibiotic exposure	φ	0.136	[−0.023, 0.294]	0.093
Antibiotic exposure—Sepsis	φ	0.221	[0.065, 0.376]	0.007
ICU—CRE colonization	φ	0.465	[0.314, 0.600]	<0.001

**Table 6:** Multivariable logistic regression of factors independently associated with carbapenem resistance (CR/CP-KP vs. CSKP/ESBL); complete-case analysis on the canonical N = 157 cohort (effective N = 150). Five prespecified covariates were entered together; CRE-associated sepsis was excluded a priori from the multivariable model owing to circularity with the case definition (reported descriptively as bivariate OR 9.90, 95% CI 3.91–25.09, *p* < 0.001 in the text). aOR = adjusted odds ratio with 95% Wald confidence interval.

Covariate	aOR (95% CI)	*p*-Value
Age 65 years or older	3.78 (1.32–10.86)	0.013
Male sex (ref = female)	0.50 (0.22–1.12)	0.093
Invasive devices	1.45 (0.64–3.27)	0.373
Recent hospitalization within 30 days	2.56 (1.16–5.63)	0.020
Current colonization	2.96 (1.24–7.05)	0.015
Model fit	AUC = 0.77; Nagelkerke R^2^ = 0.25; Hosmer–Lemeshow *p* = 0.95	Complete-case N = 150

## Data Availability

Data are contained within the article.

## Abbreviations

The following abbreviations are used in this manuscript:

aOR	Adjusted odds ratio
AST	Antimicrobial susceptibility testing
AUC	Area under the curve
BSI	Bloodstream infection
CI	Confidence interval
CP-KP	Carbapenemase-producing *K. pneumoniae*
CRE	Carbapenem-resistant Enterobacterales
CRKP	Carbapenem-resistant *K. pneumoniae*
CSKP	Carbapenem-susceptible *K. pneumoniae*
ESBL	Extended-spectrum beta-lactamase
EPV	Events per variable
EUCAST	European Committee on Antimicrobial Susceptibility Testing
ICU	Intensive care unit
IMP	Imipenemase metallo-beta-lactamase
IQR	Interquartile range
KPC	*K. pneumoniae* carbapenemase
MBL	Metallo-beta-lactamase
MDR	Multidrug-resistant
MIC	Minimum inhibitory concentration
NDM	New Delhi metallo-beta-lactamase
OR	Odds ratio
OXA-48-like	Oxacillinase-48-like carbapenemase
PCR	Polymerase chain reaction
PK/PD	Pharmacokinetic/pharmacodynamic
qSOFA	Quick Sequential Organ Failure Assessment
ROC	Receiver operating characteristic
RR	Risk ratio
SIR	Susceptible/increased exposure/resistant interpretation
SOFA	Sequential Organ Failure Assessment
TMP-SMX	Trimethoprim-sulfamethoxazole
UTI	Urinary tract infection
VAP	Ventilator-associated pneumonia
VIM	Verona integron-encoded metallo-beta-lactamase
XDR	Extensively drug-resistant

## Appendix A

**Table A1:** Complete antimicrobial susceptibility testing results, including MIC values and interpretative categories, for *K. pneumoniae* clinical isolates.

Organism/Phenotype	Class/Marker	Reported ACTIVE (S) Options	Frequently Reported R/I (From Dataset)	Notes Relevant for Discussion
*Klebsiella pneumoniae* (susceptible/non-ESBL)	ESBL-negative	Amoxicillin/clavulanate, piperacillin/tazobactam, cefuroxime, ceftriaxone, cefotaxime, ceftazidime, carbapenems (ertapenem, meropenem), aminoglycosides (amikacin, gentamicin), fluoroquinolones (ciprofloxacin, levofloxacin), TMP/SMX	Intrinsic ampicillin resistance	Typical susceptibility profile for non-ESBL *K. pneumoniae* isolates
*K. pneumoniae*—ESBL	ESBL-positive	Carbapenems (ertapenem, meropenem), generally susceptible; aminoglycosides, frequently susceptible; occasionally TMP/SMX susceptible; in some isolates: ceftazidime/avibactam, imipenem/relebactam, meropenem/vaborbactam, susceptible; occasional colistin susceptibility	Frequent resistance to third-generation cephalosporins (ceftazidime, cefepime, cefotaxime), fluoroquinolones, and sometimes piperacillin/tazobactam or amoxicillin/clavulanate	Dataset includes both classical ESBL and ESBL with additional resistance mechanisms
*K. pneumoniae*—MDR (ESBL-associated)	MDR + ESBL	Some isolates remained susceptible to meropenem; β-lactam/β-lactamase inhibitor combinations (ceftazidime/avibactam, imipenem/relebactam, meropenem/vaborbactam) active in selected isolates; amikacin frequently susceptible; colistin susceptible in several isolates	Resistance frequently observed to piperacillin/tazobactam, cefuroxime, cefotaxime, ceftazidime, ciprofloxacin, and TMP/SMX; ertapenem resistance observed in some isolates; tigecycline occasionally intermediate or resistant	Illustrates progression from ESBL phenotype toward multidrug resistance
*K. pneumoniae*—XDR	XDR	Limited active options reported: colistin, tigecycline (susceptible or intermediate), cefiderocol, aztreonam/avibactam	Resistance to nearly all remaining β-lactams, carbapenems, fluoroquinolones and other antibiotic classes; ceftazidime/avibactam resistance observed in some isolates	Highly relevant for salvage therapy discussion; synergy between ceftazidime/avibactam and aztreonam reported in selected isolates
